# Successful Surgical Treatment of Congenital Aortopulmonary Window in an Adult Patient

**DOI:** 10.1155/2011/505216

**Published:** 2011-07-27

**Authors:** Alessandro Giamberti, Raul Abella, Eduardo Consuegra Llapur, Abdallah Raweh, Silvia Cirri, Alessandro Frigiola

**Affiliations:** ^1^Association of Children with Heart Disease in the World, Milan, Italy; ^2^IRCCS Policlinico San Donato, Piazza Edmondo Malan, 20097, San Donato M.se, Milan, Italy; ^3^Hospital Universitario Materno-Infantil de Canarias, Las Palmas de GC, Canary Islands 35016, Spain; ^4^Red Crescent of United Arab Emirates, P.O. Box 3324 Abu Dhabi, UAE; ^5^Istituti Clinici Sant'Ambrogio, Via Francesco Daverio, 620122 Milan, Italy

## Abstract

Congenital aortopulmonary window is a rare inborn cardiac malformation that should be surgically treated as soon as the diagnosis is made usually during infancy. We report a successful surgical treatment of a 23-year-old male patient with a big type III aortopulmonary window.

## 1. Introduction

Congenital aortopulmonary window (APW) is a rare cardiac malformation frequently found as an isolated defect [1, 2]. Surgical repair is the treatment of choice and should be done before the network changes in pulmonary vascular have developed.

We report an interesting case of a 23-year-old male patient with a big isolated type III [3] APW that we treated during a humanitarian surgical mission in the Middle East.

## 2. Material and Methods

During a humanitarian surgical mission in the Middle East organized by the *Association of Children with Heart Disease in the World* and the *Red Crescent of United Arab Emirates*, local cardiologist presented an interesting case of congenital heart disease for eventual surgical treatment. 

A 23-year-old male patient, 68 Kg of body weight, not previously submitted cardiac surgery. He was in NYHA functional class III. The chest X-ray showed a CT index of 0.72 with signs of pulmonary overflow. The echocardiography showed an isolated 4 cm type III APW. 

The patient underwent cardiac catheterization that revealed isosystemic pressure in pulmonary arteries, a left-to-right shunt through the defect, and a Qp/Qs ratio increased from 2 to 4 after 10 minutes of 100% oxygen test.

**Figure 1:**
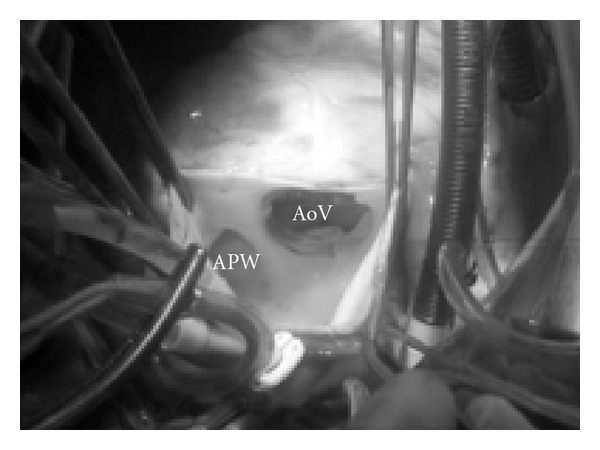
The aorta is opened anteriorly and parallel to the APW and the defect is exposed. APW: Aortopulmonary window: AoV: aortic Valve.

The surgical treatment was performed through a median sternotomy and cardiopulmonary bypass with aortic and bicaval cannulation. The pulmonary arteries were temporarily occluded to prevent pulmonary flow through the defect and to permit cardioplegia delivery to the heart. The patient was cooled to 28°C, and the aorta cross-clamped distal to the defect and the heart was arrested by cold blood cardioplegia injected into the ascending aorta. The aorta was opened anteriorly and parallel to the APW, and the defect was exposed (Figure 1) and then closed with a single heterologous pericardial patch sewn to the aortic side of the defect. The anterior aortotomy was closed with a double running polypropylene suture.

The postoperative period was uneventful. The patient was extubated 4 hours after the operation, transferred to the ward on postop day 2, and discharged from the Hospital on postop day 7.

In a follow-up period of 24 months, local cardiologists checked him twice. He is actually doing very well in NYHA functional class I without medical treatment.

## 3. Discussion

APW is a rare malformation with a physiology similar to that of patent ductus arteriosus, ventricular septal defect, and truncus arteriosus.

There is a significant arterial level systemic to pulmonary shunt with a magnitude related mainly to the size of defect and to pulmonary vascular resistance.

Surgical management is indicated at the time of diagnosis to prevent the development of irreversible pulmonary vascular disease (PVD).

Patients with a large APW usually do not survive infancy. Children or young adults with APW are occasionally encountered and frequently have marked PVD at the time of diagnosis.

Compared with the bigger surgical series [1, 2, 4, 5], our patient seems to be the oldest with APW successful submitted surgical treatment. There is one very old case reported in the literature [6]. This was a 58-year-old patient who died from a pulmonary adenocarcinoma where during autopsy, they occasionally discovered an unrepaired APW. The lung biopsy showed peripheral aneurismatic formation and thromboembolic stenosis of the lobular or segmental arteries that might provide an explanation for the long survival of this particular congenital cardiac malformation.

In our patient, since the beginning, the clinical signs, the symptoms, and the chest X-rays gave us the impression that he was still available for surgery. The cardiac catheterization confirmed our impression. The excellent postoperative course and the first 2-year followup confirmed the correct surgical indication.

We do not know why with such a big defect, he wasn't in PVD and still with a high Qp/Qs, but the rarity of the clinical and pathophysiologic picture seems to merit to be reported.

It is absolutely interesting and not rare during humanitarian surgical mission in developing countries to meet very unusual and inexplicable long-survival natural history of complex congenital heart disease.
